# Operationalising resilience in longitudinal studies: a systematic review of methodological approaches

**DOI:** 10.1136/jech-2015-206980

**Published:** 2016-08-08

**Authors:** T D Cosco, A Kaushal, R Hardy, M Richards, D Kuh, M Stafford

**Affiliations:** MRC Unit for Lifelong Health and Ageing, London, UK

**Keywords:** AGEING, Epidemiological methods, Research Design in Epidemiology

## Abstract

Over the life course, we are invariably faced with some form of adversity. The process of positively adapting to adverse events is known as ‘resilience’. Despite the acknowledgement of 2 common components of resilience, that is, adversity and positive adaptation, no consensus operational definition has been agreed. Resilience operationalisations have been reviewed in a cross-sectional context; however, a review of longitudinal methods of operationalising resilience has not been conducted. The present study conducts a systematic review across Scopus and Web of Science capturing studies of ageing that posited operational definitions of resilience in longitudinal studies of ageing. Thirty-six studies met inclusion criteria. Non-acute events, for example, cancer, were the most common form of adversity identified and psychological components, for example, the absence of depression, the most common forms of positive adaptation. Of the included studies, 4 used psychometrically driven methods, that is, repeated administration of established resilience metrics, 9 used definition-driven methods, that is, a priori establishment of resilience components and criteria, and 23 used data-driven methods, that is, techniques that identify resilient individuals using latent variable models. Acknowledging the strengths and limitations of each operationalisation is integral to the appropriate application of these methods to life course and longitudinal resilience research.

## Introduction

Over the life course, we are invariably faced with some form of adversity. Responses to adversity are diverse, ranging from very negative, for example, psychiatric disorder and premature mortality, to very positive, for example, thriving, and may be physiological, psychological or social in nature. The process of positively adapting to adverse events is known as ‘resilience’.^1^ ^2^ Despite the acknowledgement of two common components of resilience, that is, adversity and positive adaptation, no consensus operational definition has been agreed.

Owing to the unobservable nature of the construct, resilience cannot physically be measured, only inferred via measurement of its two constituent components.^3^ Consequently, there are several ways in which these components can be operationalised to identify resilient individuals. Three popular means of operationally defining resilience in longitudinal studies are psychometrically driven, definition-driven and data-driven methods.

The majority of studies to date have examined resilience in cross-sectional studies.^4–6^ Longitudinal studies capture at least three waves of data and are able to provide data that illuminate trends that occur over time.^7^ Many variables are not static, interacting dynamically and changing over time; therefore, longitudinal methods must be employed to disentangle these relationships. Consequently, these studies provide greater insights into the nature of a phenomenon than is possible with cross-sectional methods or two-wave pre–post follow-up designs.^7^

Longitudinal studies that employ psychometrically driven methods repeatedly administer previously validated resilience scales such as the widely used Connor-Davidson Resilience Scale.^8^ These methods have been developed under the assumption that resilience is a universal concept that can be operationalised uniformly across populations and age groups using a single scale. Thresholds may be applied to identify resilient individuals, but generally resilience is captured on a continuum. Whereas the definition-driven and data-driven approaches to longitudinal data are used to identify resilient individuals based on dynamic measures of adaptation, repeat observations of resilience captured by psychometric scales are used to describe continuity or change in resilience over time.

Definition-driven methods use an a priori set of criteria and components to establish which individuals are resilient. The adversity and adaptation components included in these definitions, and the thresholds used to establish which individuals are resilient, are usually determined by the researchers; generally there is no established benchmark. Within a longitudinal context, resilience may involve the continued avoidance or absence of a negative state, for example, symptoms of depression. In contrast to psychometrically driven methods, definition-driven methods are situation-specific, that is, thresholds are applied within the specific adversity–adaptation dyad examined in a given study.

Data-driven methods are used to identify resilient groups of people or levels of resilience on a continuum using statistical procedures. These methods generally employ latent variable models, such as growth mixture modelling (GMM). GMM is a person-centred latent variable modelling procedure that allows the identification of subgroups with similar outcome trajectories in samples with at least three repeated-measure data collection waves.^9^ Within the framework of resilience, individuals who function physically, mentally or socially particularly well over time, despite experiencing some sort of adversity, for example, cancer, can be identified as ‘resilient’. As with definition-driven methods, data-driven methods are specific to the adversity–adaptation dyad.

Although there have been two reviews of cross-sectional resilience metrics and measurement,^5^ ^6^ a review of longitudinal methods of operationalising resilience has not been conducted. The aim of the current study is to systematically review studies of ageing to examine the ways in which resilience has been operationalised in longitudinal studies to deepen our understanding of how to maximise resilience in the challenges faced by an ageing population. Through an investigation of the ways in which adverse events and positive adaptations are used in resilience operationalisations, we aim to identify practical methods for characterising resilient individuals. It is hoped that by providing a comprehensive snapshot of the ways in which resilience has been operationalised, clinicians, policymakers and researchers will be better informed as to how to apply and critically evaluate these models in their own work.

## Methods

### Search strategy

A systematic review was conducted across Scopus (which provides 100% MEDLINE, Embase and Compendex coverage) and Web of Science databases. Between 5 February 2015 and 11 February 2015, the search terms ‘resilience AND (ageing OR aging)’ were employed. In Scopus, article title, abstract and keywords were searched across all years. In Web of Science, ‘topics’ were searched across all years excluding books, letters, corrections, meetings or editorial, that is, non-peer reviewed articles. Additionally, reference lists and relevant articles were hand searched.

### Inclusion criteria

Studies were included in the final analysis if they met the following criteria: (1) original peer-reviewed research, (2) operationally defined resilience, for example, quantified resilience using individual data and (3) the study was longitudinal, that is, collected at least three waves of quantitative data.^7^

### Exclusion criteria

Studies were excluded if they met the following criteria: (1) ineligible article type, that is, conference proceeding, editorial, commentary, perspective, book chapter, book review and dissertation; (2) non-English article; (3) resilience beyond or below the level of the individual, for example, family or cellular resilience and (4) resilience as a personality trait, for example, overcontroller, undercontroller and resilient personality types.^10^

### Screening

TDC, MS and AK conducted independent title/abstract and full-text screening. Disagreements concerning the decision to include studies in the data extraction phase were resolved via discussion.

### Data extraction

Demographics, that is, age, gender distribution, sample population and study characteristics, were extracted from the included studies. Information regarding the components of resilience, that is, positive adaption, adverse event, as well as the analytical methods for quantifying resilience, for example, data-driven approach using GMM, were also collected.

## Results

### Search

**Figure 1:**
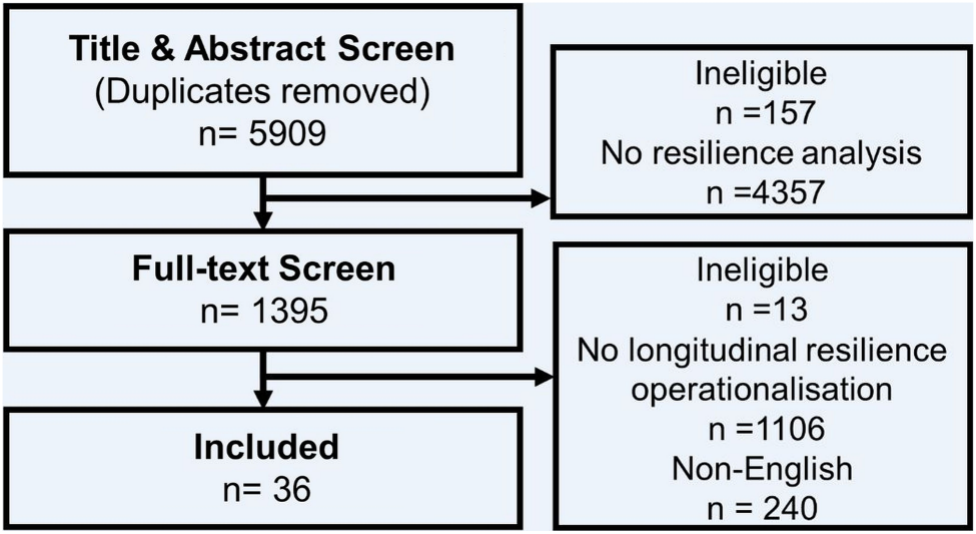
Study inclusion flow chart.

We were interested only in studies of individual-level resilience but did not identify suitable search terms to exclude studies of resilience at higher and lower level units at the title/abstract screening stage. Furthermore, we did not limit the search to studies with resilience in the results sections of articles since this also had the potential to miss relevant studies. Thus, a large number of articles (5909) were yielded at this stage. Of these, 36 met inclusion criteria (figure 1). Although there are related and potentially overlapping terms, such as resistance and adaptation, we limited our search to the specific term of resilience used by the original authors.

### Included studies

**Figure 2:**
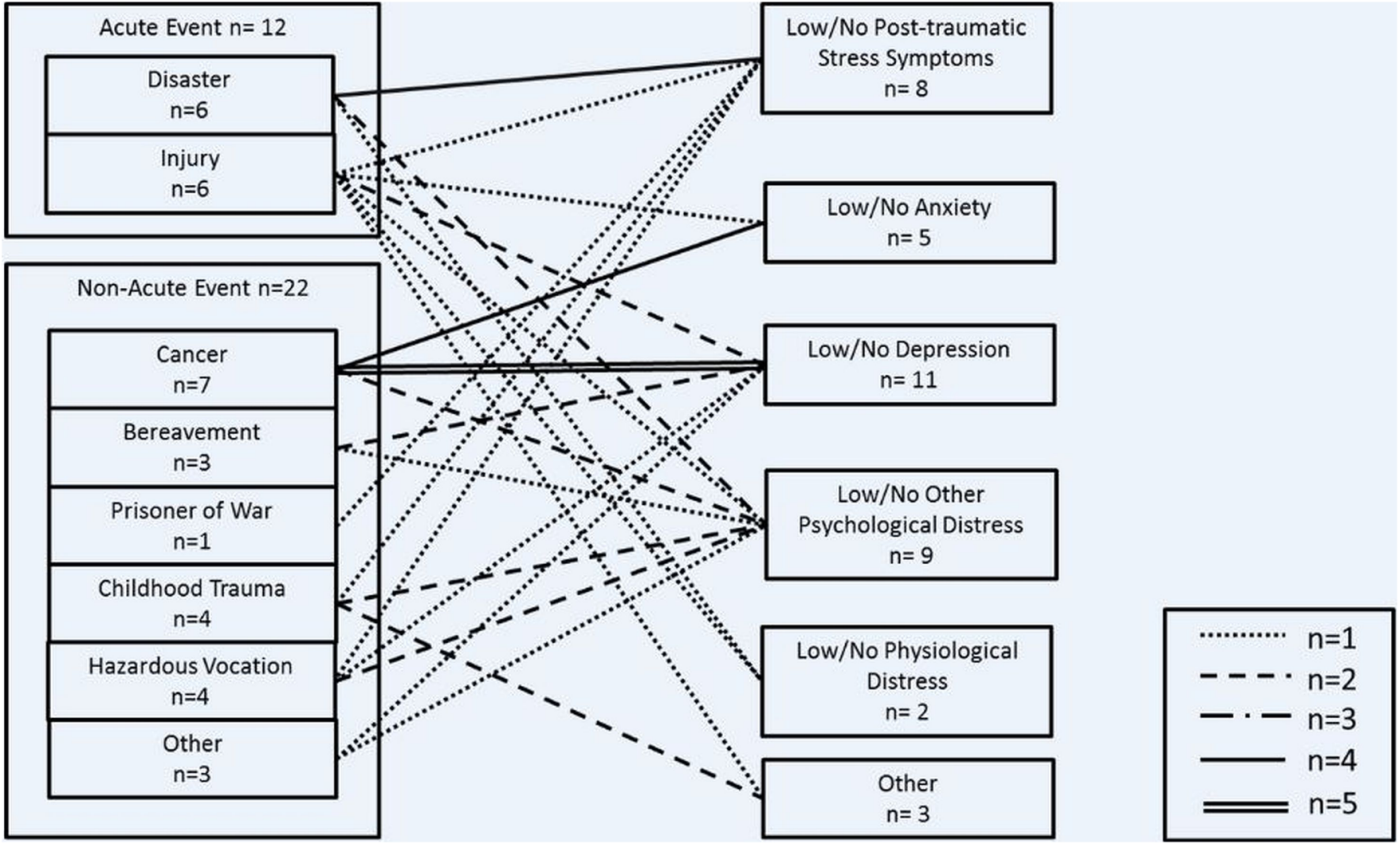
Adversity and positive adaptation relationships in included studies.

Included studies (n=36) most commonly examined protective/risk factors for resilience and were conducted in the USA (n=16) with young-aged to middle-aged adults, that is, 20–40 years (table 1). Sample size ranged from 30 to 10 835 with an average of 758.69 (SD=1877.6) and median of 233.5. Studies conducted a minimum of three waves of data collection and a maximum of seven (mean=3.9; SD=3.9), with an average follow-up period of 265.4 weeks (SD=461.4 weeks). The source of adversity varied greatly; more studies included non-acute adversity, for example, cancer, than acute adversity, for example, disaster. The positive adaptations to these adverse events were less varied, generally demonstrated by low levels of psychological distress, for example, low levels of anxiety or post-traumatic stress symptoms (figure 2).

**Table 1:** Included study demographic characteristics

Study	n	Age (years)				Follow-up		Country	Female (%)	Population
		Minimum	Maximum	Mean	SD	Data collection waves	Length (weeks)			
Psychometrically driven										
Donohoe *et al*^11^	33	13	14			3	12	Scotland	24.2	Secondary school children
Fortney *et al*^12^	30			40.5	10.1	4	36	USA	60.0	Primary care clinicians
Ritchie *et al*^13^	73	12	18			3	52	Canada		First Nation youth
Songprakun and McCann^14^	56	18	58	42.1	9.7	3	12	Thailand	73.2	Psychiatric outpatients
Definition-driven										
Boe *et al*^15^	70			34.7	9.3	4	1274	Norway	0.0	Disaster survivors
Bonanno *et al*^16^	185	65		72	6.5	3	72	USA		Bereaved spouses
Bonanno *et al*^17^	185	65		72	6.5	3	72	USA		Bereaved spouses
Ho *et al*^18^	76	21	66	38.9	9.2	4	52	China		Hereditary gastrointestinal cancer registry
Jaffee^19^	2065	8	16	10.96	4.54	3	144		54.0	Maltreated children
Mlinac *et al*^20^	470			79.9	5.8	4	192	USA	74.9	Community-dwelling older adults
Netuveli *et al*^21^	3581	50				3	Varied	UK	57.2	Community-dwelling older adults
Solomon *et al*^22^	64					3	1820	Israel		Veterans; ex-POWs
Werner^4^	49					4	936	USA		Offspring of alcoholics
Data-driven										
Bonanno and Mancini^23^ ^24^	997			42	14	3	52	China	61.0	SARS epidemic survivors
Bonanno *et al*^25^	233					4	104	Austria, Germany, Ireland, Sweden, Switzerland, UK	21.90	Spinal cord injury
deRoon-Cassini *et al*^26^	330			40.4	15.8	4	24	USA		Traumatic injury patients
Dunn *et al*^27^	398					6	24	USA	100.0	Breast cancer surgery patients
Dunn *et al*^28^	252					7	26	USA	53.6	Oncology patients; family caregivers
Galatzer-Levy *et al*^29^	234	21	43	27.42	4.78	4	208	USA	15.4	Police officers
Galatzer-Levy *et al*^30^	234	21	43	27.42	4.78	4	208	USA	15.4	Police officers
Holgersen *et al*^31^	70					4	1404	Norway	0.0	Disaster survivors
Hou *et al*^32^	234	29	82	64.44	10.55	4	52	China	38.0	Colorectal cancer
Lam *et al*^33^	285			50.6	10.1	4	32	China	100.0	Breast cancer patients
Lam *et al*^34^	186			56.2	9.1	4	32	China	100.0	Breast cancer survivors
Larm *et al*^35^	1432			16.5	1.47	4	1300	Sweden	33.8	Clinical substance abuse; general population
Le Brocque *et al*^36^	190	6	16	10.7	2.31	3	24	Australia	37.0	Accident victims
Murphy and Marelich^37^	111	6	11	8.5	1.8	4	72	USA	45.9	Children of HIV/AIDS diagnosed mothers
Norris *et al*^38^ ^39^	561					4	72	Mexico		Flood victims
	1267					4	120	USA		
Nugent *et al*^40^	201	7	18	12	3	4	144	USA		Children referred to Family Advocacy Program
Pietrzak *et al*^41^	10 835			45.3	9.6	3	416	USA	13.4	9/11 responders
Saad *et al*^42^	398					6	24	USA	100.0	Breast cancer surgery patients
Self-Brown *et al*^43^	426	8	16	11.63	2.26	5	100	USA	51	Hurricane Katrina survivors
Sterling *et al*^44^	155	18	69	36.9	12.8	4	52	Australia	63	Whiplash patients
Sveen *et al*^45^	95	19	89	44.7	15.5	3	52	Sweden	24.2	Burn victims
Tang *et al*^46^	447			48.9	12.6	4	25	Taiwan	67.8	Caregivers of terminal patients
Zhu *et al*^47^	2172	45	65			4	312	USA	67.0	Chronic pain

POW, prisoner of war; SARS, severe acute respiratory syndrome.

### Methods of operationalisation

The majority (n=23) of studies conducted data-driven operationalisation procedures, followed by definition-driven (n=9) and psychometrically driven (n=4) methods. One study used psychometrically driven and definition-driven methods,^20^ that is, using a definition to capture a group of resilient individuals and then examining the level of resilience later in these groups using the resilience scale.^48^

Psychometrically driven methods repeatedly employed an established resilience scale: Donohoe *et al*^11^ repeatedly administered the Prince-Embury Resiliency Scale for Children and Adolescents,^49^ and Fortney *et al*,^12^ Songprakun and McCann^14^ and Mlinac *et al*^20^ repeatedly administered the resilience scale.^48^

Definition-driven methods generally included the maintenance of an adaptive state throughout the duration of the study, demonstrated by lower levels of mental health problems, notably depression, than might be expected in the face of adversity. For example, in a study of bereaved spouses, resilient individuals were those who demonstrated low or no depression throughout 18 months of follow-up^16^ (table 2). Within the data-driven methods, several person-centred latent variable techniques, that is, statistical procedures used to group similar individuals based on a common unobserved variable, were employed: latent class analysis (n=1), longitudinal hierarchical clustering (n=2), semiparametric group-based clustering (n=3) and GMM (n=17) (table 3). GMM, the most popular method, is a specific form of latent variable modelling that allows the identification of classes, or groupings of individuals with similar trajectories, based on individuals' scores on a continuous variable over a number of waves of data collection. Researchers are able to classify individuals as belonging to a specific trajectory based on the similarity of their slopes and intercepts. For example, in a study of individuals with spinal cord injury, GMM was employed to identify a group of individuals who demonstrated low levels of depression over the duration of the study.^32^ Latent class analysis, longitudinal hierarchical clustering and semiparametric group-based clustering use similar approaches to GMM, that is, using latent variable models to identify groups of individuals based on similar longitudinal patterns.

**Table 2:** Definition-driven study characteristics

Study	Adversity	Adaptation	Subsample	Prevalence of resilience (%)
Boe *et al*^15^	Disaster	No PTSD		58.3
Bonanno *et al*^16^*	Spousal bereavement	No or low† depression		45.9
Bonanno *et al*^17^*	Spousal bereavement	No or low† depression		45.9
Ho *et al*^18^	Hereditary cancer risk	Below HADS threshold of 7/8	HADS—anxiety	66.7
			HADS—depression	76.8
Jaffee^19^	Childhood maltreatment	Meet or exceed national norms for mental health, academic achievement and social competence		37–49
Mlinac *et al*^20^	External stressors or life events common to late life	Coaches felt that participants met their goals despite more significant stressors		28.6
Netuveli *et al*^21^	Functional limitation, bereavement, marital separation, poverty	Return to preadversity GHQ scores postadversity		14.3
Solomon *et al*^22^	War veterans	No PTSD	Control veterans	88.8
			ex-POWs	26.6
Werner^4^	Offspring of alcoholics	No coping problems at age 18		59.2

*Same data set used.

†<80th centile z-scores on the Center for Epidemiologic Studies—depression scale.^50^

A prototypical resilience trajectory, that is, decreasing functioning followed by a return to pre-event functioning, was also identified.^38^

GHQ, General Health Questionnaire; HADS, Hospital Anxiety and Depression Scale;^51^ POWs, prisoners of war; PTSD, post-traumatic stress disorder.

**Table 3:** Data-driven study characteristics

Study	Adversity (population*)	Positive adaptation	Trajectory model†	Prevalence of resilience (%)
Bonanno *et al*^23^ ^24^	SARS epidemic survivors	High psychological and physical functioning		35.0
Bonanno *et al*^25^	Spinal cord injury	Low anxiety	Anxiety (unconditional model)	57.5
			Anxiety (conditional model)	58.1
		Low depression	Depression (unconditional model)	66.1
			Depression (conditional model)	50.8
deRoon-Cassini *et al*^26^	Traumatic injury patients	Low depression		58.0
Dunn *et al*^27^	Breast cancer surgery patients	Low depression/anxiety		38.9
Dunn *et al*^28^	Oncology patients; family caregivers	Low depression		56.3
Galatzer-Levy *et al*^29^‡	Police officers	Low psychological distress		76.7
Galatzer-Levy *et al*^30^‡	Police officers	Low psychological distress		76.7
Holgersen *et al*^31^	Disaster survivors	Positive mental health		61.4
Hou *et al*^32^	Colorectal cancer	No depression/anxiety		65–37
Lam *et al*^33^‡	Breast cancer patients	Low psychological distress		66.0
Lam *et al*^34^‡	Breast cancer survivors	Low psychological distress		66.0
Larm *et al*^35^	Clinical substance abuse; general population		High resilience in GP	52.4
			Good resilience in GP	47.6
			High resilience in CS	24.4
			High to moderate resilience in CS	24.5
			Moderate to high resilience in CS	33.0
			Low to moderate resilience in CS	9.3
			Low resilience in CS	8.8
Le Brocque *et al*^36^	Accident victims	Few PTSD symptoms		57.0
Murphy and Marelich^37^	Children of HIV/AIDS diagnosed mothers	Cognitive function, externalising behaviours, social skills		32.4
Norris *et al*^38^ ^39^	Mexican flood victims	Few PTSD symptoms		32.0
	9/11 New York residents	Few PTSD symptoms		10.1
Nugent *et al*^40^	Children referred to Family Advocacy Program	Few PTSD symptoms		60.7
Pietrzak *et al*^41^	9/11 responders	Few PTSD symptoms		58.0
Saad *et al*^42^	Breast cancer surgery patients	Low depression/anxiety		38.9
Self-Brown *et al*^43^	Hurricane Katrina survivors	Few PTSD symptoms		71.0
Sterling *et al*^44^	Whiplash patients	Low neck disability		40.0
Sveen *et al*^45^	Burn victims	No PTSD		40.0
Tang *et al*^46^	Caregivers of terminal patients	Low depression		11.4
Zhu *et al*^47^	Chronic pain	Low depression		72.5

*Samples were taken from populations exposed to adversity.

†Trajectory models where one or more resilience trajectories are identified.

‡Same data set used.

CS, clinical population sample; GP, general population sample; PTSD, post-traumatic stress disorder; SARS, severe acute respiratory syndrome.

## Discussion

Data-driven methods, notably latent variable models, were the most commonly used methods for operationalising resilience in longitudinal studies of ageing. Non-acute events were the most common source of adversity and the absence of psychological distress over time the most prominent source of positive adaptation. However, positive adaptation has primarily been measured by the absence of psychopathology and there have been no studies specifically measuring positive mental adaptation and well-being.

Several limitations must be acknowledged in the interpretation of these results. The present study intends to provide a comprehensive overview of methods used to capture resilience in studies that have specifically used the term ‘resilience’. Similar phrases or terms used by authors that may have intended to capture resilience, for example, hardiness or resistance, would not have been included in the present study. This may apply more to biomedically oriented disciplines where the term resilience is not as embedded in the description of responses to adversity as it is in psychologically oriented disciplines. In addition to the general resilience term, there are a number of modifiers that may be added to specify a particular form of resilience, for example, family resilience and biological resilience. In the interest of making direct comparisons of resilience operationalisations, only studies that specifically used the term ‘resilience’ as a standalone construct were included. Consequently, this may have prevented the inclusion of other forms of resilience and predisposed the positive adaption variables towards psychological outcomes. Although the majority of studies captured in this review examined protective factors for resilience, an analysis of these factors has not been included due to the heterogeneity of adversity/adaptation dyads and operationalisation methods. Protective factors are likely specific to the particular definition and therefore are not necessarily generalisable across all resilience definitions.

Psychometrically driven models of resilience used previously established, continuous measures of resilience. These models have primarily been used in cross-sectional studies and the resilience scales used have demonstrated adequate psychometric properties;^5^ ^6^ however, four studies in the present review used these metrics longitudinally. Of note, these studies did not have resilience as their primary focus, but rather used resilience as one of many outcome variables. These methods are effective in that they capture a continuous measure of resilience using previously validated psychometrics and permit a high level of granularity (ie, ability to provide detailed information). For existing studies that include resilience scales and for prospective studies, this is an effective means of operationalising resilience; however, these operationalisations are not possible for researchers using secondary data sets that have not previously administered these scales.

To date, there has not been a longitudinal analysis of resilience using an established metric where resilience is the primary outcome of interest; studies have not yet examined the ways in which resilience changes and interacts with events or behaviours. Factors that shape resilience in different stages of life and the relationship of future resilience with past resilience have not been explored in the literature, which is dominated by cross-sectional research. Prospective longitudinal studies that have the capacity to disentangle these relationships will provide invaluable information on the ways in which resilience exists across the life course.

Definition-driven methods are the simplest and most easily employed methods of longitudinally operationalising resilience. These methods generally stipulated the continued absence of a negative outcome, for example, depression, during or after experiencing a negative event. More complex definitions were also identified, for example, different thresholds for subsequent waves of follow-up, as well as the development of a priori prototypical resilience trajectories.^23^ ^38^ Prototypical resilience trajectories posited a decrease in functioning at the onset of an adverse event followed by a return to pre-event levels of functioning.^38^ This is an improvement on steady-state definitional models of resilience, as longitudinal aspects of resilience are acknowledged and included in a dynamic model. These methods can be applied in any circumstance in which an adversity–adaptation dyad using categorical or continuous variables exists, which is advantageous for researchers using secondary data. Where possible, clinically derived or previously validated cut-offs are recommended in the classification of adaptation–adversity dyads.

Shortcomings of definition-driven methods include impediments to granularity and generalisability. In studies using a binary threshold, a large degree of granularity is lost. This can be particularly problematic in longitudinal studies with older adults where individuals are unable to uphold optimal states of functioning in a binary model.^52^ Given the context-specific nature of definitions, these methods do not have a high degree of generalisability. In existing secondary data sets, the application of specific resilience definitions is limited to the variables captured in the study. This is problematic for longstanding longitudinal studies that have been collecting data for many years, but have not employed a resilience scale. Furthermore, in the absence of established benchmarks, researchers may use different thresholds for resilience limiting cross-study comparisons.

Data-driven methods employed statistical procedures to identify groups of individuals as resilient. Given that resilience cannot be directly measured, latent variable modelling techniques were employed, the most popular of these being GMM. Latent variable modelling is a meritorious method of identifying resilient individuals due to the removal of researcher-defined thresholds, that is, greater objectivity, and the ability to categorise individuals into different relative trajectories. In contrast to definition-driven methods that employ a series of components and thresholds, latent variable modelling allows group membership to be determined based on the characteristics of individuals in the sample relative to each other rather than relative to an external criterion. This is useful in unpicking different levels of resilience using person-centred methods, that is, study participants with similar performances, rather than variable-centred methods, that is, participants who perform above or below an a priori threshold on a variable, as in definition-driven methods. Studies in the present review generally captured three waves of data over an average of 5 years; however, when more follow-up data waves are available, data-driven methods are better able to represent changing trajectories than definition-driven methods that posit binary states. Therefore, in circumstances with many repeat waves of data collection with continuous variables, data-driven methods are recommended over definition-driven methods in the articulation of resilience.

Several caveats must be acknowledged in the identification of resilience using GMM and other latent variable techniques. First, the identification of trajectories, although informed by objective fit indices, for example, Bayesian Information Criteria, are interpreted by the author. Other factors, such as fit to theoretical underpinnings, are also taken into account and balanced against fit indices; the final model selection is at the discretion of the author. Furthermore, the identification of trajectories is conducted only using individuals in a given sample with a specific set of demographic and cohort attributes, producing a set of trajectories specific to the study. As such, the cross-study generalisability of these methods is low.

In the identification of trajectories, the researcher dubs the trajectory as ‘resilient’ or not based on their subjective interpretation of the slope and intercept of the trajectory. Consequently, a researcher may choose to dub a trajectory ‘high functioning’ or ‘resistant’ rather than ‘resilient’ due to personal preference rather than conceptual differences. Although strides towards consensus resilience trajectory shapes have been made, through the use of definition-driven a priori prototypical trajectories,^38^ ^53^ these trajectories are not necessarily employed nor do they necessarily marry with results from latent variable analyses.

The methods captured in the present review operationalise resilience using three different methods: psychometrically driven, definition-driven and data-driven. Psychometrically driven methods are generalisable, continuous measures of resilience that are applicable across studies. These studies, however, require that a resilience scale has been repeatedly administered in a study, which inhibits analysis in data sets that have not collected these data, for example, pre-existing longitudinal studies. Definition-driven methods employ situation-specific thresholds for continuous and categorical adaptation–adversity dyads. To date, these models have had low granularity due to the application of binary models and many have demonstrated limited generalisability due to study-specific constituent components of resilience and thresholds used. Data-driven methods employ person-centred statistical procedures to group similar individuals, using the granularity of continuous variables. These methods provide a level of objective classification; however, the subjectivity of model fit interpretation and situation-specific nature of the trajectories inhibits generalisability. Continued refinement of longitudinal resilience research concepts and methods, for example, through the inclusion of life course perspectives, will provide greater insights into the dynamic nature of positive adaptations to adverse events.

**What is already known on this subject**

- Resilience involves positively adapting to adverse events. The majority of resilience research has been conducted in cross-sectional studies. Longitudinal studies provide greater insights into the nature of a phenomenon than is possible with cross-sectional methods or two-wave pre–post follow-up designs.

**What this study adds**

- The present study systematically reviews methods for operationalising resilience in longitudinal studies. Extant methods are synthesised and critically examined, highlighting their strengths and limitations for future research.

## References

[1] Luthar SS, Doernberger CH, Zigler E Resilience is not a unidimensional construct: insights from a prospective study of inner-city adolescents. Dev Psychopathol 1993;5:703–17. 10.1017/S0954579400006246 25722542 PMC4339070

[2] Rutter M Resilience in the face of adversity. Protective factors and resistance to psychiatric disorder. Br J Psychiatry 1985;147:598–611. 10.1192/bjp.147.6.598 3830321

[3] Luthar SS Vulnerability and resilience—a study of high-risk adolescents. Child Dev 1991;62:600–16. 10.2307/1131134 1914628 PMC4235608

[4] Werner EE Resilient offspring of alcoholics—a longitudinal-study from birth to age-18. J Stud Alcohol 1986;47:34–40. 10.15288/jsa.1986.47.34 3959559

[5] Windle G, Bennett KM, Noyes J A methodological review of resilience measurement scales. Health Qual Life Outcomes 2011;9:8 10.1186/1477-7525-9-8 21294858 PMC3042897

[6] Ahern NR, Kiehl EM, Sole ML, et al A review of instruments measuring resilience. Issues Compr Pediatr Nurs 2006;29:103–25. 10.1080/01460860600677643 16772239

[7] Singer JD, Willett JB Applied longitudinal data analysis: modeling change and event occurrence. Oxford (NY): Oxford University Press, 2003.

[8] Connor KM, Davidson JR Development of a new resilience scale: the Connor-Davidson Resilience Scale (CD-RISC). Depress Anxiety 2003;18:76–82. 10.1002/da.10113 12964174

[9] Jung T, Wickrama K An introduction to latent class growth analysis and growth mixture modeling. Soc Personal Psychol Compass 2008;2:302–17. 10.1111/j.1751-9004.2007.00054.x

[10] Block J Lives through time. Berkeley (CA): Bancroft, 1971.

[11] Donohoe C, Topping K, Hannah E The impact of an online intervention (Brainology) on the mindset and resiliency of secondary school pupils: a preliminary mixed methods study. Educ Psychol 2012;32:641–55. 10.1080/01443410.2012.675646

[12] Fortney L, Luchterhand C, Zakletskaia L, et al Abbreviated mindfulness intervention for job satisfaction, quality of life, and compassion in primary care clinicians: a pilot study. Ann Fam Med 2013;11:412–20. 10.1370/afm.1511 24019272 PMC3767709

[13] Ritchie SD, Wabano MJ, Russell K, et al Promoting resilience and wellbeing through an outdoor intervention designed for Aboriginal adolescents. Rural Remote Health 2014;14:2523. 24670144

[14] Songprakun W, McCann TV Effectiveness of a self-help manual on the promotion of resilience in individuals with depression in Thailand: a randomised controlled trial. BMC Psychiatry 2012;12:12 10.1186/1471-244X-12-12 22339984 PMC3298500

[15] Boe HJ, Holgersen KH, Holen A Reactivation of posttraumatic stress in male disaster survivors: the role of residual symptoms. J Anxiety Disord 2010;24:397–402. 10.1016/j.janxdis.2010.02.003 20207518

[16] Bonanno GA, Wortman CB, Lehman DR, et al Resilience to loss and chronic grief: a prospective study from preloss to 18-months postloss. J Pers Soc Psychol 2002;83:1150–64. 10.1037/0022-3514.83.5.1150 12416919

[17] Bonanno GA, Wortman CB, Nesse RM Prospective patterns of resilience and maladjustment during widowhood. Psychol Aging 2004;19:260–71. 10.1037/0882-7974.19.2.260 15222819

[18] Ho SM, Ho JW, Bonanno GA, et al Hopefulness predicts resilience after hereditary colorectal cancer genetic testing: a prospective outcome trajectories study. BMC Cancer 2010;10:279 10.1186/1471-2407-10-279 20537192 PMC2891641

[19] Jaffee SR Sensitive, stimulating caregiving predicts cognitive and behavioral resilience in neurodevelopmentally at-risk infants. Dev Psychopathol 2007;19:631–47. 10.1017/S0954579407000326 17705896 PMC3709833

[20] Mlinac M, Lees F, Stamm K, et al Maintaining late life health behaviors comparing clinician rating and self-reported resilience. Top Geriatr Rehabil 2014;30:188–94. 10.1097/TGR.0000000000000021

[21] Netuveli G, Wiggins RD, Montgomery SM, et al Mental health and resilience at older ages: bouncing back after adversity in the British Household Panel Survey. J Epidemiol Community Health 2008;62:987–91. 10.1136/jech.2007.069138 18854503

[22] Solomon Z, Horesh D, Ein-Dor T, et al Predictors of PTSD trajectories following captivity: a 35-year longitudinal study. Psychiatry Res 2012;199:188–94. 10.1016/j.psychres.2012.03.035 22486946

[23] Bonanno GA, Mancini AD The human capacity to thrive in the face of potential trauma. Pediatrics 2008;121:369–75. 10.1542/peds.2007-1648 18245429

[24] Bonanno GA, Ho SM, Chan JC, et al Psychological resilience and dysfunction among hospitalized survivors of the SARS epidemic in Hong Kong: a latent class approach. Health Psychol 2008;27:659–67. 10.1037/0278-6133.27.5.659 18823193

[25] Bonanno GA, Kennedy P, Galatzer-Levy IR, et al Trajectories of resilience, depression, and anxiety following spinal cord injury. Rehabil Psychol 2012;57:236–47. 10.1037/a0029256 22946611

[26] deRoon-Cassini TA, Mancini AD, Rusch MD, et al Psychopathology and resilience following traumatic injury: a latent growth mixture model analysis. Rehabil Psychol 2010;55:1–11. 10.1037/a0018601 20175629

[27] Dunn LB, Cooper BA, Neuhaus J, et al Identification of distinct depressive symptom trajectories in women following surgery for breast cancer. Health Psychol 2011;30:683–92. 10.1037/a0024366 21728421 PMC3217116

[28] Dunn LB, Aouizerat BE, Langford DJ, et al Cytokine gene variation is associated with depressive symptom trajectories in oncology patients and family caregivers. Eur J Oncol Nurs 2013;17:346–53. 10.1016/j.ejon.2012.10.004 23187335 PMC4114773

[29] Galatzer-Levy IR, Brown AD, Henn-Haase C, et al Positive and negative emotion prospectively predict trajectories of resilience and distress among high-exposure police officers. Emotion 2013;13:545–53. 10.1037/a0031314 23339621 PMC3974969

[30] Galatzer-Levy IR, Steenkamp MM, Brown AD, et al Cortisol response to an experimental stress paradigm prospectively predicts long-term distress and resilience trajectories in response to active police service. J Psychiatr Res 2014;56:36–42. 10.1016/j.jpsychires.2014.04.020 24952936 PMC5759781

[31] Holgersen KH, Klockner CA, Boe HJ, et al Disaster survivors in their third decade: trajectories of initial stress responses and long-term course of mental health. J Trauma Stress 2011;24:334–41. 10.1002/jts.20636 21594899

[32] Hou WK, Law CC, Yin J, et al Resource loss, resource gain, and psychological resilience and dysfunction following cancer diagnosis: a growth mixture modeling approach. Health Psychol 2010;29:484–95. 10.1037/a0020809 20836603

[33] Lam WWT, Bonanno GA, Mancini AD, et al Trajectories of psychological distress among Chinese women diagnosed with breast cancer. Psychooncology 2010;19:1044–51. 20014074 10.1002/pon.1658

[34] Lam WWT, Shing YT, Bonanno GA, et al Distress trajectories at the first year diagnosis of breast cancer in relation to 6 years survivorship. Psychooncology 2012;21:90–9. 10.1002/pon.1876 21132676

[35] Larm P, Hodgins S, Tengstrom A, et al Trajectories of resilience over 25 years of individuals who as adolescents consulted for substance misuse and a matched comparison group. Addiction 2010;105:1216–25. 10.1111/j.1360-0443.2010.02914.x 20331546

[36] Le Brocque RM, Hendrikz J, Kenardy JA The course of posttraumatic stress in children: examination of recovery trajectories following traumatic injury. J Pediatr Psychol 2010;35:637–45. 10.1093/jpepsy/jsp050 19541598

[37] Murphy DA, Marelich WD Resiliency in young children whose mothers are living with HIV/AIDS. AIDS Care 2008;20:284–91. 10.1080/09540120701660312 18351474 PMC2422847

[38] Norris FH, Tracy M, Galea S Looking for resilience: understanding the longitudinal trajectories of responses to stress. Soc Sci Med 2009;68:2190–8. 10.1016/j.socscimed.2009.03.043 19403217

[39] Norris FH, Tracy M, Galea S Looking for resilience: understanding the longitudinal trajectories of responses to stress. Soc Sci Med 2009;68:2190–8. 10.1016/j.socscimed.2009.03.043 19403217

[40] Nugent NR, Saunders BE, Williams LM, et al Posttraumatic stress symptom trajectories in children living in families reported for family violence. J Trauma Stress 2009;22:460–6. 10.1002/jts.20440 19718758

[41] Pietrzak RH, Feder A, Singh R, et al Trajectories of PTSD risk and resilience in World Trade Center responders: an 8-year prospective cohort study. Psychol Med 2014;44:205–19. 10.1017/S0033291713000597 23551932

[42] Saad S, Dunn LB, Koetters T, et al Cytokine gene variations associated with subsyndromal depressive symptoms in patients with breast cancer. Eur J Oncol Nurs 2014;18:397–404. 10.1016/j.ejon.2014.03.009 24726621 PMC4074554

[43] Self-Brown S, Lai BS, Thompson JE, et al Posttraumatic stress disorder symptom trajectories in Hurricane Katrina affected youth. J Affect Disord 2013;147:198–204. 10.1016/j.jad.2012.11.002 23206321 PMC4231137

[44] Sterling M, Hendrikz J, Kenardy J Compensation claim lodgement and health outcome developmental trajectories following whiplash injury: a prospective study. Pain 2010;150:22–8. 10.1016/j.pain.2010.02.013 20307934

[45] Sveen J, Ekselius L, Gerdin B, et al A prospective longitudinal study of posttraumatic stress disorder symptom trajectories after burn injury. J Trauma 2011;71:1808–15. 10.1097/TA.0b013e31822a30b8 21841510

[46] Tang ST, Huang GH, Wei YC, et al Trajectories of caregiver depressive symptoms while providing end-of-life care. Psychooncology 2013;22:2702–10. 10.1002/pon.3334 23760787

[47] Zhu Z, Galatzer-Levy IR, Bonanno GA Heterogeneous depression responses to chronic pain onset among middle-aged adults: a prospective study. Psychiatry Res 2014;217:60–6. 10.1016/j.psychres.2014.03.004 24679514 PMC4122231

[48] Wagnild GM, Young HM Development and psychometric evaluation of the Resilience Scale. J Nurs Meas 1993;1:165–78. 7850498

[49] Prince-Embury S, Steer RA Profiles of personal resiliency for normative and clinical samples of youth assessed by the resiliency scales for children and adolescents (TM). J Psychoeduc Assess 2010;28:303–14. 10.1177/0734282910366833

[50] Radloff LS The CES-D scale: a self-report depression scale for research in the general population. Appl Psychol Meas 1977;1:385–401. 10.1177/014662167700100306

[51] Zigmond AS, Snaith RP The hospital anxiety and depression scale. Acta Psychiatr Scand 1983;67:361–70. 10.1111/j.1600-0447.1983.tb09716.x 6880820

[52] Cosco TD, Stephan B, Brayne C (Unsuccessful) binary modeling of successful aging in the oldest-old adults: a call for continuum-based measures. J Am Geriatr Soc 2014;62:1597–8. 10.1111/jgs.12958 25116987

[53] Bonanno GA, Moskowitz JT, Papa A, et al Resilience to loss in bereaved spouses, bereaved parents, and bereaved gay men. J Pers Soc Psychol 2005;88:827–43. 10.1037/0022-3514.88.5.827 15898878

